# Neuromas cause severe residual problems at long-term despite surgery

**DOI:** 10.1038/s41598-023-42245-4

**Published:** 2023-09-21

**Authors:** Emma Dahlin, Hanna Gudinge, Lars B. Dahlin, Erika Nyman

**Affiliations:** 1 Department of Biomedical and Clinical Sciences, Linköping University, Linköping, Sweden; 2 Department of Translational Medicine-Hand Surgery, Lund University, Jan Waldenströms gata 5, 20502 Malmö, Sweden; 3 Varberg Hospital, Region Halland, Varberg, Sweden; 4 Department of Hand Surgery, Skåne University Hospital, Malmö, Sweden; 5 Department of Hand Surgery, Plastic Surgery and Burns, Linköping University Hospital, Linköping, Sweden

**Keywords:** Neurology, Trauma

## Abstract

Pain, and disabilities after neuroma surgery, using patient reported outcome measurements (PROMs), were evaluated by QuickDASH and a specific Hand Questionnaire (HQ-8). The 69 responding individuals (response rate 61%; 59% women; 41% men; median follow up 51 months) reported high QuickDASH score, pain on load, cold sensitivity, ability to perform daily activities and sleeping difficulties. Individuals reporting impaired ability to perform daily activities and sleeping problems had higher scores for pain, stiffness, weakness, numbness/tingling, cold sensitivity and QuickDASH. Only 17% of individuals reported no limitations at all. No differences were observed between sexes. Surgical methods did not influence outcome. Symptoms and disabilities correlated moderately-strongly to each other and to ability to perform regular daily activities as well as to sleeping difficulties. Pain, cold sensitivity, sleeping difficulties and limitation to perform daily activities were associated to higher QuickDASH. A weak association was found between follow up time and QuickDASH score as well as pain on load, but not cold sensitivity. A major nerve injury was frequent among those with limitations during work/performing other regular daily activities. Despite surgical treatment, neuromas cause residual problems, which affect the capacity to perform daily activities and ability to sleep with limited improvement in long-term.

## Introduction

A neuroma may emerge after an unrepaired or inappropriately repaired or reconstructed nerve injury^1,2^. Neuromas may remain asymptomatic, but a few can cause substantial problems and even develop to a chronic pain syndrome^3^ with impaired ability to perform daily activities and to sleep properly^4^. Of all new cases with chronic pain that appear every year, 26.7/100.000 of them are caused by iatrogenic or traumatic nerve injuries^5^. The incidence of symptomatic neuromas is, however, hard to estimate^3^, while incidence of nerve injuries is estimated to 13.9 (15.21 for amputations) per 100,000 person-year; being more frequent among men and younger persons^6^. It has been reported that about 50% of individuals with a nerve injury may suffer from chronic pain, where 73% of these may have neuropathic pain^7^. Symptomatic neuroma after amputation in lower and upper limbs of 4.2% and 25%, respectively, have been reported^8^.

Painful neuromas can be treated by medical or surgical methods. Surgical methods include transposition of the neuroma away from exposed painful region into a suitable tissue or a material as well as repair or reconstruction of the nerve injury or defect to make the nerve fibres regenerate into the distal nerve end with possibility to regain function^1,9–15^; the former being described as more frequently used^11,16–21^. Targeted muscle reinnervation (TMR) is a novel technique to treat neuroma^22–24^ providing a pathway for axonal outgrowth limiting a disorganized growth pattern forming a neuroma. Neurolysis, or decompression, with or without coverage of a neuroma-in-continuity with flaps or tissue, is also utilized^25,26^. A common indication for neuroma surgery is neuropathic pain after a nerve injury, which should be related to a risk of depression or sleeping problems, affecting wellbeing^27,28^. The number of surgical procedures for neuroma treatment, and severity of preoperative and postoperative pain, have an impact on the Disability of the Arm, Shoulder and Hand (DASH) score^29^. The choice of surgical method may also have an impact to lower disability score and improve depression and quality of life^27,29^.

Patient related outcome measurements (PROMs), including QuickDASH and the eight specific questions related to hand symptoms and ability (HQ-8), can be used to evaluate symptoms and disability after surgery^30^. Remaining pain problems and its relation to general activities of daily living as well as sleeping problems associated to different pain modalities have not sufficiently been highlighted after neuroma surgery^31^, in which there are also risks for long sick-leave and unemployment^32^.

Our aim was, by using two validated questionnaires for patient rated outcome, to evaluate remaining symptoms and disability as well as to analyse the relation between pain modalities, activities of daily living, ability to sleep and other symptoms in individuals having surgery for neuroma in the upper limb in a long time follow up.

## Materials and methods

### Study design

**Figure 1:**
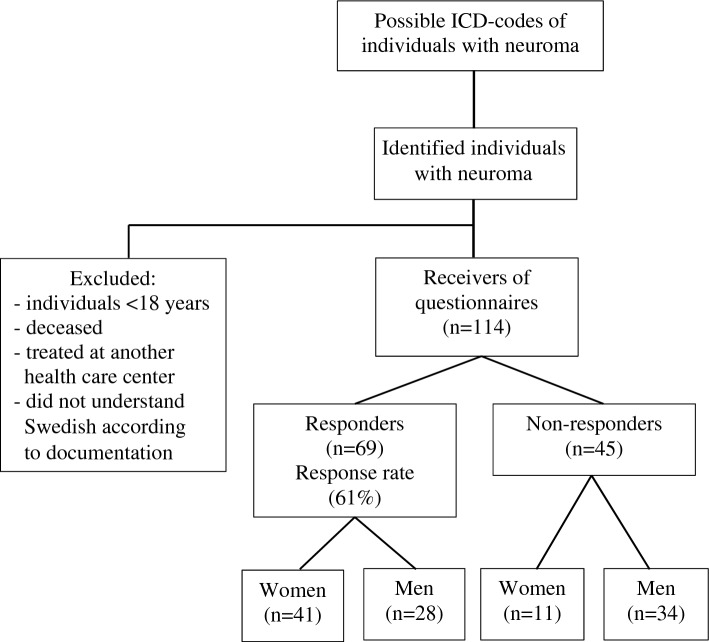
Overview of the study population with surgically treated neuroma in the upper limb.

Individuals ≥ 18 years, surgically treated for neuroma in the upper limb January 1st 2008 to June 30th 2020, at the Department of Hand Surgery, Plastic Surgery, and Burns, University Hospital of Linköping, Sweden and the Department of Hand Surgery, Skåne University Hospital, Sweden were identified, using appropriate ICD-10 codes, and included (Fig. 1). If individuals were treated at another health care centre or were documented to not understand Swedish, they were excluded. Data on individual characteristics, were collected from medical charts by two of the researchers (ED and HG), who did not participate in the treatment of the individuals. All identified individuals were treated for a single neuroma.

A short form of the Disability of Arm, Shoulder and Hand questionnaire (QuickDASH)^33^ and a validated specific Hand Questionnaire (HQ-8)^30^, consisting of eight questions, were sent out to the individuals together with written information about the study and a consent form. Non-responders were reminded once by another postal letter, as well as they were contacted by telephone. Questionnaires were answered in the late autumn of 2020. The HQ-8 questionnaire contains questions on the individuals’ experience in the affected hand/arm; pain at rest, pain on motion without load, pain on load, stiffness, weakness, numbness/tingling in fingers, cold sensitivity, and ability to perform daily activities. Values are presented on a scale 0–100, divided into tens (0, 10, 20… 100, 0 = no problems, 100 = worse possible problems). The questions “During the past week, how much difficulty have you had sleeping because of the pain in your arm, shoulder or hand?” and “During the past week, were you limited in your work or other regular daily activities as a result of arm, shoulder or hand problem” were selected from the QuickDASH survey. These two questions are coded from 1 to 5 where 1 = no problems and 5 = extensive problems.

### Ethics

Ethical approval was provided after application by the Swedish Ethical Review Authority (registry number 2020-01484 0617). Informed consent was received from each subject that replied to the questionnaires. All methods were performed in accordance with relevant guidelines and regulations, including the Declaration of Helsinki.

### Statistical methods

Nominal data are presented as number (%). Continuous data are presented as median [interquartile range] and compared using the Mann–Whitney U-test. Categorical variables are compared using the Chi-square test (Fisher´s exact test if n < 5 in a group). A Spearman’s correlation test between age at follow up, QuickDASH, and HQ-8 variables was conducted. A p-value of < 0.05 was accepted as significant. A rho-value of ≥ 0.30 was required for any correlation (0.3–0.7 = moderate and > 0.7 = strong correlation). Linear regression analyses were performed to study the independent variables individual characteristics (i.e., sex, age at surgery, smoking, type of nerve, iatrogenic nerve injury), and surgical method (i.e., nerve transposition and nerve suture/reconstruction) on pain at rest and pain on load as well as on QuickDASH (dependent variables; model 1). The presence of sleeping difficulties, and ability to perform daily activity were also used as independent variables to examine any effect on the two dependent pain modalities and QuickDASH (dependent; model 2). In addition, linear regression analyses were performed to evaluate if presence of cold sensitivity had any association to pain at rest and pain on load (model 3). Finally, in model 3 it was also evaluated if the two pain modalities influenced the total QuickDASH score. A linear regression analysis was also done to investigate any association between total QuickDASH, the pain modalities, and cold sensitivity (dependent variables) and follow up time (independent variable), adjusted for age, sex, and surgical methods (i.e., nerve transposition and nerve suture/reconstruction). Data was collected, coded, and analysed in the program IBM SPSS Statistics, version no 28 (Armonk, USA).

## Results

### Characteristics of the population

Individual characteristics are presented in Table 1. Questionnaires were sent out to 114 individuals and the response rate was 61%. Responders and non-responders differed significantly regarding sex (p < 0.001) and age at time of surgery (p = 0.049), where women and individuals at higher age responded more frequently.

**Table 1:** Basic characteristics of individuals surgically treated for a neuroma in the upper limb.

	Responders^a^ n = 69 (61)
Age at surgery (years)	50 [34–57]
Sex (women/men)	41 (59)/28 (41)
Smoking (yes/no)	15 (22)/53 (77)
Injured nerve	
Median nerve	10 (15)
Ulnar nerve	7 (10)
Radial nerve	22 (32)
Digital nerve	23 (33)
Musculocutaneous nerve	1 (1)
Accessory nerve	2 (3)
Medial cutaneous antebrachial nerve	4 (6)
Type of nerve	
Sensory	51 (74)
Motor	7 (10)
Mixed	11 (16)
Initial nerve injury mechanism	
Nerve transection	63 (91)
Amputation	4 (6)
Crush injury	2 (3)
Cause of injury	
Iatrogenic	33 (48)
Home equipment	16 (23)
Professional tools	13 (19)
Animal bite	4 (6)
Explosions	1 (1)
Self-inflicted injury	2 (3)

Values are median [IQR; i.e., 25th–75th percentiles] or n (%). ^a^Responders were more often women and older (p < 0.001 and p = 0.049, respectively). No other differences between responders and non-responders were found.

### Postoperative QuickDASH and HQ-8 scores

**Figure 2:**
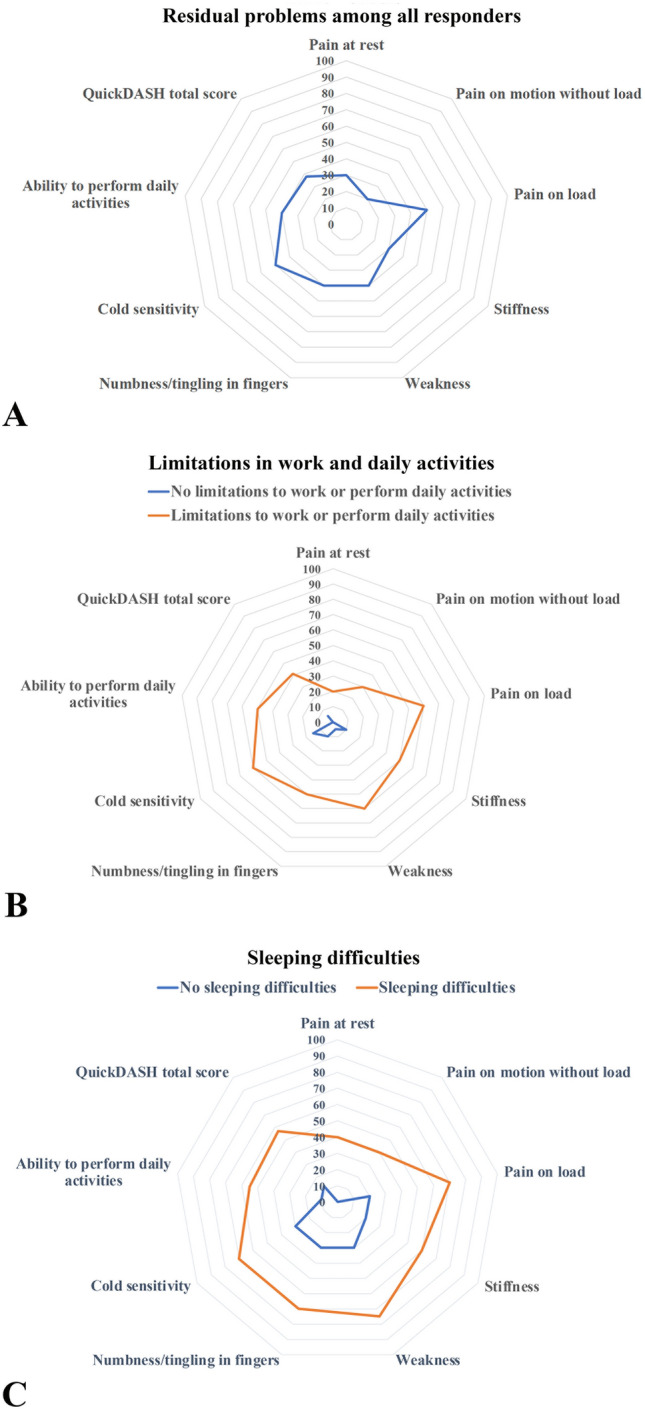
Spider diagrams showing residual problems after surgery for neuroma among all responders (**a**), as well as those with and without limitations in work or daily activities (**b**) and sleeping difficulties (**c**). Spider diagrams showing the results of total QuickDASH and H-Q8 scores among all responders (**a**), among individuals having and not having limitations in work or in daily activities (**b**) and among individuals not having and having sleeping difficulties (**c**). The limitations were based on the QuickDASH question “During the past week, were you limited in your work or other regular daily activities because of your arm, shoulder, or hand problem?” Individuals who were slightly, moderately, very limited or unable to work or to perform daily activities were included in the group “limitations”. In accordance, the definition of sleep problems was based on the question in QuickDASH as no sleeping difficulties or the other categories.

Among the individuals responding to the two questionnaires at long time follow up (51 [22–103] months), pain of different modalities, related symptoms, impaired ability to perform daily activities, limitations in work or other regular daily activities as well as sleeping problems were described to a variable extent (Fig. 2a). However, no significant differences were seen between women and men (Table 2).

**Table 2:** Results from QuickDASH and HQ-8 questionnaires divided by sex among individuals surgically treated for a neuroma in the upper limb.

	Total population n = 69	Women n = 41	Men n = 28	P-value
Questionnaire—time to follow up after surgery (months)	51 [22–103]	47 [22–94]	54 [17–108]	0.75
Total QuickDASH score	38 [12–57]	41 [22–57]	32 [7–57]	0.46
Limited in work or other regular daily activities (QuickDASH)				0.71
Not limited at all	12 (17)	5 (12)	7 (25)	
Slightly limited	13 (19)	8 (20)	5 (18)	
Moderately limited	24 (35)	15 (37)	9 (32)	
Very limited	10 (15)	7 (17)	3 (11)	
Unable	10 (15)	6 (15)	4 (14)	
Difficulties to sleep (QuickDASH)				0.63
No difficulty	35 (51)	20 (49)	15 (54)	
Mild difficulty	13 (19)	10 (24)	3 (11)	
Moderate difficulty	9 (13)	5 (12)	4 (14)	
Severe difficulty	7 (10)	3 (7)	4 (14)	
So much that I can’t sleep at all	5 (7)	3 (7)	2 (7)	
Pain (HQ-8)				
At rest	20 [0–40]	20 [0–45]	10 [0–40]	0.764
On motion without load	20 [ 0–50]	30 [5–50]	10 [0–48]	0.64
On load	50 [20–70]	60 [25–80]	40 [10–70]	0.22
Stiffness (HQ-8)	30 [10–65]	40 [20–65]	20 [3–68]	0.57
Weakness (HQ-8)	40 [20–80]	50 [25–75]	40 [10–80]	0.79
Numbness/tingling in fingers (HQ-8)	40 [20–70]	40 [30–65]	40 [10–80]	0.91
Cold sensitivity (HQ-8)	50 [10–80]	40 [10–75]	55 [20–90]	0.29
Ability to perform daily activities (HQ-8)	40 [5–60]	50 [30–60]	35 [0–58]	0.09

Values are median [IQR; i.e., 25th–75th percentiles] or n (%). P-values based on Mann–Whitney U-test (continuous data) or Chi-squared test (or Fisher´s exact test if n < 5 in a group; nominal data, independent samples; sex). QuickDASH and HQ-8 questionnaires were sent to individuals’ post-surgery.

QuickDASH The Quick Disabilities of the Arm, Shoulder and Hand (DASH) questionnaire is a self-administered region-specific outcome instrument developed as a measure of self-rated upper limb disability and symptoms, HQ-8 Hand Surgery Questionnaire used in national quality register from Sweden.

### Characteristics and symptoms of individuals with and without limitation to work or perform other regular daily activities

Among the individuals, 12 (17%) individuals did not report any limitations to work or to perform other regular daily activities, while 57 (83%) were slightly, moderately, very limited or had no ability to work or to perform other regular daily activities. No statistically significant differences were found concerning basic characteristics, such as age at follow up, age at surgery, sex, surgical method, or response on questionnaire at time from surgery for the two categories of individuals with or without limitations to work or to perform other regular daily activities (Table 3).

**Table 3:** Characteristics, symptoms, and disabilities, based on QuickDASH and HQ-8 questionnaires among individuals surgically treated for a neuroma in the upper limb with and without limitations in work or ability to perform daily activities.

	No limitations to work or perform other regular daily activities (QuickDASH question no 8) n = 12 (17)	Slightly, moderately, very limited or unable to work or perform other regular daily activities (QuickDASH question no 8) n = 57 (83)	P-value
Age at surgery (years)	57 [32–65]	49 [35–56]	0.22
Sex			0.17
Women	5 (42)	36 (63)	
Men	7 (58)	21 (37)	
Injured nerve			**0.007**
Major nerve	4 (33)	42 (74)	
Digital nerve	8 (67)	15 (26)	
Surgical methods			0.25
Nerve transposition^a^	3 (30)	26 (50)	
Nerve repair/reconstruction^b^	7 (70)	26 (50)	
Total QuickDASH score	5 [1–7]	41 [24–68]	** < 0.001**
Difficulties to sleep (QuickDASH)			
No difficulty	12 (100)	23 (40)	**0.002**
Mild difficulty	0 (0)	13 (23)	
Moderate difficulty	0 (0)	9 (16)	
Severe difficulty	0 (0)	7 (12)	
So much that I can’t sleep at all	0 (0)	5 (9)	
Pain (HQ-8)			
At rest	0 [0–0]	20 [10–50]	** < 0.001**
On motion without load	0 [0–8]	30 [10–50]	** < 0.001**
On load	0 [0–28]	60 [30–80]	** < 0.001**
Stiffness (HQ-8	10 [0–20]	50 [20–70]	** < 0.001**
Weakness (HQ-8)	5 [0–18]	60 [30–80]	** < 0.001**
Numbness/tingling in fingers (HQ-8)	10 [0–38]	50 [30–80]	**0.002**
Cold sensitivity (HQ-8)	15 [0–30]	60 [20–90]	**0.002**
Ability to perform daily activities (HQ-8)	0 [0–0]	50 [30–65]	** < 0.001**

Significant values are marked in bold.

Values are median [IQR; i.e., 25th–75th percentiles] or n (%). P-values based on Mann–Whitney U-test (continuous data) or Chi-squared test (nominal data, independent samples; sleeping difficulties).

^a^Transposition, with and without conduits.

^b^Excision and suturing, autograft, allograft, repair with conduits.

Others excluded n = 7.

However, several significant differences were identified between the limitation group and the non-limitation group regarding the type of injured nerve, where the presence of a major nerve injury was more frequent among those with limitations to work or to perform other regular daily activities. There was a significantly higher total QuickDASH score in those with limitations. Significant differences were also seen regarding difficulties to sleep, pain at rest, pain on motion without load, pain on load, weakness, numbness/tingling, cold sensitivity and the ability to perform daily activities (Table 3; Fig. 2b).

### Characteristics, symptoms, and ability to perform daily activities among individuals with and without sleeping problems

Thirty-four out of 69 individuals (49%) described sleeping difficulties at the follow up, but there were no significant differences concerning sex, age at follow up, age at surgery, injured nerve or response on questionnaire at time from surgery (Table 4). However, there was a significant difference in the use of surgical method between the groups of no sleeping difficulties and sleeping difficulties, where individuals with sleeping difficulties were more often surgically treated with nerve transposition compared to other procedures (Table 4). Significant differences were found concerning the pain modalities, where individuals with sleeping problems reported higher pain scores at rest, on motion without load as well as on load. The individuals with sleeping problems also reported higher scores concerning stiffness, weakness, numbness/tingling and cold sensitivity. They also described a higher score in ability to perform daily activities, limitations in work or other regular daily activities and a significantly higher total QuickDASH score (Table 4; Fig. 2c).

**Table 4:** Characteristics, symptoms and disabilities, based on QuickDASH and HQ-8 questionnaires, among individuals surgically treated for a neuroma in the upper limb with and without sleeping difficulties.

	No sleeping difficulties (QuickDASH question no 11) n = 35 (51)	Sleeping difficulties (QuickDASH question no 11) n = 34 (49)	P-value
Age at surgery (years)	55 [41–65]	54 [42–61]	0.68
Sex			0.81
Women	20 (57)	21 (62)	
Men	15 (43)	13 (38)	
Injured nerve			0.13
Major nerve^a^	20 (57)	26 (77)	
Digital nerve	15 (43)	8 (24)	
Surgical procedures^b^			**0.03**
Nerve transposition^c^	10 (35)	19 (57)	
Nerve repair/reconstruction^d^	19 (66)	14 (42)	
Pain (HQ-8)			
At rest	0 [0–10]	40 [20–63]	** < 0.001**
On motion without load	0 [0–10]	40 [30–63]	** < 0.001**
On load	20 [0–40]	70 [50–80]	** < 0.001**
Stiffness (HQ-8)	20 [0–30]	60 [30–83]	** < 0.001**
Weakness (HQ-8)	30 [10–40]	75 [58–90]	** < 0.001**
Numbness/tingling (HQ-8)	30 [0–40]	70 [40–80]	** < 0.001**
Cold sensitivity (HQ-8)	30 [0–50]	70 [38–90]	** < 0.001**
Ability to perform daily activities (HQ-8)	10 [0–40]	55 [40–80]	** < 0.001**
Limited in work or other regular daily activities			
Not limited at all	12 (34)	0 (0)	** < 0.001**
Slightly limited	10 (29)	3 (9)	
Moderately limited	11 (31)	13 (38)	
Very limited	1 (3)	9 (27)	
Unable	1 (3)	9 (27)	
Total QuickDASH score	13 [5–30]	57 [41–76]	** < 0.001**

Significant values are marked in bold.

Values are median [IQR; i.e., 25th–75th percentiles] or n (%). P-values based on Mann–Whitney U-test (continuous data) or Chi-squared test (nominal data, independent samples; sleeping difficulties).

^a^Median nerve, ulnar nerve, radial nerve, accessory nerve, musculocutaneus nerve or medial cutaneous brachial nerve.

^b^All procedures except ‘’others’’.

^c^Transposition with and without conduits.

^d^Excision of neuroma and nerve repair with sutures, repair with conduits or nerve reconstructions with autograft or allograft.

### Characteristics, symptoms, and disabilities grouped by surgical procedure

Nerve transposition and excision of neuroma with a nerve repair or nerve reconstruction were generally observed in an equal number of individuals [29 (47%) and 33 (53%) individuals, respectably] (Table 5). There were no statistically significant differences between these two main groups of surgical procedures, concerning age at follow up, age at surgery, questionnaire time to follow up after surgery, sex, injured nerve, limitations in work or other regular daily activities, QuickDASH total score, pain, stiffness, weakness, numbness/tingling, cold sensitivity, or ability to perform daily activities (Table 5).

**Table 5:** Characteristics, symptoms/and disabilities (based on QuickDASH and HQ-8 questionnaires) of individuals with a surgically treated neuroma in the upper limb grouped by surgical procedure.

	Nerve transposition n = 29 (47)	Excision of neuroma and repair or nerve reconstruction n = 33 (53)	P-value
Age at surgery (years)	51 [34–60]	45 [27–56]	0.29
Questionnaire response—time since surgery (months)	47 [21–104]	56 [22–94]	0.98
Sex			0.54
Women	17 (59)	20 (61)	
Men	12 (41)	13 (39)	
Injured nerve			0.46
Major nerve^a^	18 (62)	22 (67)	
Digital nerve	11 (38)	11 (33)	
Pain (HQ-8)			
At rest	20 [10–50]	10 [0–40]	0.51
On motion without load	30 [10–50]	10 [0–45]	0.38
On load	60 [30–75]	30 [10–70]	0.17
Stiffness (HQ-8)	30 [10–55]	30 [15–70]	0.40
Weakness (HQ-8)	50 [25–75]	40 [15–80]	0.75
Numbness/tingling in fingers (HQ-8)	50 [30–75]	40 [15–65]	0.61
Cold sensitivity (HQ-8)	70 [30–90]	30 [10–65]	0.07
Ability to perform daily activities (HQ-8)	50 [30–65]	40 [0–50]	0.16
Difficulties to sleep			
No difficulty	10 (35)	19 (58)	0.53
Mild difficulty	8 (28)	5 (15)	
Moderate difficulty	6 (21)	3 (9)	
Severe difficulty	5 (17)	2 (6)	
So much that I can’t sleep at all	0 (0)	4 (12)	
Limited in work or other regular daily activities			
Not limited at all	3 (10)	7 (21)	0.38
Slightly limited	5 (17)	7 (21)	
Moderately limited	11 (38)	11 (33)	
Very limited	7 (24)	2 (6)	
Unable	3 (10)	6 (18)	
Total QuickDASH score	39 [22–63]	32 [11–58]	0.53

Values are median [IQR; i.e., 25th–75th percentiles] or n (%). P-values based on Mann–Whitney U-test (continuous data) or Chi-squared test (nominal data, independent samples; surgical procedure).

^a^Median nerve, ulnar nerve, radial nerve, accessory nerve, musculocutaneus nerve or medial cutaneous brachial nerve.

### Correlations

A Spearman correlation test was performed to analyse the correlations between age of the individuals at follow up, the replies from the HQ-8 questionnaire as well as the questions limitations in work or other regular daily activities, sleeping difficulties and total score from the QuickDASH questionnaire. There were no correlations found among age at follow up and different variables in the questionnaires. There were significant positive moderate to strong correlations between all the evaluated variables (Table 6).

**Table 6:**
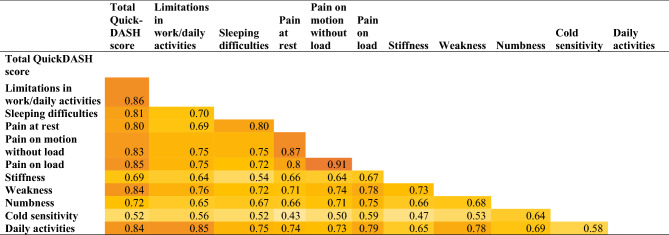
Rho-values from correlation analyses of symptoms and disabilities, based on the questionnaires QuickDASH and HQ-8 at follow up, among individuals surgically treated for a neuroma in the upper limb.

Values are rho-values (Spearman correlation test). P-values for the rho values are required as 0.005 after correction with Bonferroni. All rho-values being significant. Only rho-values ≥ 0.3 presented (i.e., 0.3–0.7 = moderate and > 0.7 = strong correlation).

The postoperative follow up time did weakly and negatively correlate with pain on load (r = − 0.31, p = 0.009), but not with total QuickDASH (r < 0.30), pain at rest (p > 0.05) or cold sensitivity (p > 0.05).

### Regression analyses

The factors age at surgery, sex, smoking, type of nerve, iatrogenic injury or not or surgical methods did not affect pain at rest or on load or total QuickDASH in the linear regression analysis (model 1). In contrast, the dependent variable pain at rest was predicted by presence of sleeping. Pain on load as well as total QuickDASH were predicted by both the presence of sleeping difficulties and limitations in work or to perform daily activities (model 2). Cold sensitivity predicted a higher score of both pain at rest and on load. Linear regression analysis, with total QuickDASH score as dependent variable, indicated that pain at rest and pain on load predicted a higher QuickDASH score (model 3; Table 7). Postoperative follow up time was associated with a weak association with the total QuickDASH score (− 0.2 [− 0.3 to − 0.2]; p = 0.03) and pain on load − 0.2 [− 0.4 to − 0.1]; p = 0.009), but not with pain at rest (− 0.1 [− 0.3 to 0.1]; p = 0.17) or cold sensitivity (− 0.1 [− 0.3 to 0.1]; p = 0.16). Adjustment with or without the factor surgical method did not affect the values.

**Table 7:** Regression analyses of the basic individual characteristics, some HQ-8 questions and total QuickDASH score among individuals surgically treated for a neuroma in the upper limb.

Independent variables	Model 1 Dependent			Model 2 Dependent			Model 3 Dependent		
	Pain at rest	Pain on load	QuickDASH	Pain at rest	Pain on load	QuickDASH	Pain at rest	Pain on load	QuickDASH
Age at surgery	0 [− 0.4–0.4] *0.96*	0 [− 0.6–0.4] *0.71*	0 [− 0.4–0.4] *0.99*	Adjusted	Adjusted	Adjusted	Adjusted	Adjusted	Adjusted
Sex (women reference)	3 [− 11–18] *0.65*	− 8 [− 24–9] *0.35*	− 3 [− 18–11] *0.64*	Adjusted	Adjusted	Adjusted	Adjusted	Adjusted	Adjusted
Smoker (no smoker reference)	0 [0–0] *0.39*	0 [0–0] *0.12*	0 [0–0] *0.21*	–	–	–	–	–	–
Type of nerve (digital nerve reference)	10 [− 5–25] *0.19*	6 [− 11–23] *0.48*	− 9 [− 6–24] *0.26*	–	–	–	–	–	–
Iatrogenic nerve injury (other causes reference)	− 12 [− 16–13] *0.82*	9 [− 17–16] *0.97*	− 1 [− 15–14] *0.90*	–	–	–	–	–	–
Transposition (nerve repair/reconstruction reference)	3 [− 7–12] *0.57*	10 [0–21] *0.06*	5 [− 5–14] *0.31*	–	–	–	–	–	–
Sleep disturbances (no sleep disturbances reference)	–	–	–	**33** **[21–45] < *0.001***	**33** **[20–46] < *0.001***	**27** **[18–36] < *0.001***	–	–	–
Limitations in activity (no limitations reference)	–	–	–	10 [− 2–23] *0.10*	**22** **[9–36] *0.002***	**25** **[16–35] < *0.001***	–	–	–
Cold sensitivity	–	–	–	–	–	–	**0.4** **[0.2–0.5] < *0.001***	**0.5** **[0.4–0.7] < *0.001***	–
Pain at rest	–	–	–	–	–	–	–	–	**0.4** **[0.2–0.6] < *0.001***
Pain on load	–	–	–	–	–	–	–	–	**0.5** **[0.3–0.6] < *0.001***

Significant values are marked in bold.

Values are unstandardized B [CI 95%] *p-value.* Adjusted = in the analysis in the model adjusted for age and sex.

## Discussion

The present study has shown that individuals surgically treated for neuroma in the upper limb may have extensive remaining postoperative symptoms and disabilities, such as different pain modalities, impaired ability to perform daily activities, limitations in work and other regular daily activities as well as sleeping problems. The present median follow up time is a rather long-term considering neuroma related problems^5,34,35^. The problems did not differ between women and men, neither did the outcome differ between active and passive surgical procedures. Individuals that were limited to work or to perform other regular daily activities had more symptoms and disabilities as well as more often difficulties to sleep. Surgery on major nerves yielded more limitations compared to procedures on digital nerves. Moderate to strong correlations were seen between all the HQ-8 variables and the QuickDASH variables without any correlation to age. Sleeping problems and limitations to activities were associated with more pain, symptoms, and disabilities. Furthermore, cold sensitivity was associated with more pain at rest and on load, while the latter also were associated with more symptoms and disabilities.

Individuals with surgically treated neuroma may have postoperative symptoms and disabilities, also probably depending on the extent of the primary injury as well as the possibility for tissue reconstruction, which affects their ability to work, to perform regular daily activities as well as the ability to sleep. Earlier studies have shown that surgical treatment of neuroma can improve patient reported pain^27,36,37^, including improvements of QuickDASH and pain scores after treatment with a bioresorbable nerve caping device^38,39^. However, there is no guarantee that all individuals will be helped by the surgical intervention^3^, which is indicated by others authors^5,34,40^. Half of the present population reported sleeping difficulties with an association to more pain problems and extensive symptoms and disabilities indicated by a higher total QuickDASH score. Sleeping difficulties in relation to postoperative neuroma problems have not been emphasized in earlier studies, but is an important aspect after treatment, because it is shown to be related to different pain modalities, other symptoms as well as the ability to work. It might be possible to treat sleeping difficulties related to neuropathic pain with a low dose of tricyclic antidepressant, e.g., amitriptyline, before bedtime or using a variety of other strategies^41^. Whether symptoms of depression were present among the individuals are unknown since no specific questionnaire revealing any signs of depression or anxiety was used in the present study. Although, depression can be related to a higher age and more pain interference^42^. Furthermore, it has been described that surgical treatment of a neuroma can improve the patient reported depression score^27^. Individuals with neuroma are also at risk for longer opioid use, where surgery can reduce the use of such drug in individuals^28^. However, long-term use of opioids and gabapentinoid drugs (e.g., gabapentin or pregabalin) are not known among individuals with neuroma irrespective of surgery being performed or not.

Some of the present individuals experienced substantial symptoms, disabilities, and limitations after surgical treatment at the same level as individuals with nerve compression disorders before surgery^43^, particularly in the presence of neuropathic pain^44^, but individuals with nerve compression disorders usually have a substantial relief of symptoms and disabilities after surgery^43^. We did not observe any significant differences regarding any variables among women and men, while women with a nerve compression syndrome report more cold sensitivity^45^ and a higher QuickDASH score after surgery for ulnar nerve compression at elbow^43^. In addition, neuropathic pain is more frequent in subjects older than 60 years than in younger subjects, and in women than in men, as well as is more severe than non-neuropathic pain^46^.

The surgical method did not appear to influence or to be associated with outcome, which differs from a previous study, reporting that neuroma excision and nerve repair resulted in lower disability scores than transposition or simple excision^29^. A nerve transposition into a vein, compared to nerve transposition into a muscle, has also been reported to cause less pain, a better sensory function as well as improved function^1^. Burying of a neuroma in the superficial branch of the radial nerve into the brachioradialis muscle is reported to be more efficient than burying elsewhere^5,47^. A recent systematic review and meta-analysis reports that target muscle reinnervation (TMR)^22,23^ shows promising results in surgical treatment of neuroma^34^, particularly for the superficial branch of the radial nerve^24^. Experimental data indicates that long acellular nerve allografts, without a distal nerve end connected, limit neuroma formation^48^, which is in accordance with published data on alterations in neurophysiological properties, such as ongoing activity, after rat sciatic nerve regeneration in a mesothelial chamber^49^. However, no larger human studies in the upper limb have approached the use of “blind” nerve allografts in neuroma treatment^50,51^, but treatment with a “blind” nerve cap improves pain and function^38,39^. We cannot state anything about the efficacy of different old, or novel, transposition techniques to surgically treat neuroma, since no comparison of different transposition methods was possible or done in the present study. Another point is that superficially located neuroma, for example at wrist level or in a digital nerve in a finger, may induce more symptoms than a deeply located nerve due to the bolstering effect and tissue vascularisation of the latter. Furthermore, such nerves are subjected also to higher risk for traumatic injuries, irrespective of the cause, due to its anatomical localization. These superficially located nerves are mainly sensory nerve branches, which cause more residual pain problems compared to motor nerves, reflected by the low number of motor nerves in the present study. Moreover, injured motor nerves may have higher capacity of recovery if surgically treated with nerve repair or reconstruction or with tendon transfer^52^. Finally, a nerve repair should not be performed with tension^53^; a number of alternatives are available (e.g., nerve conduits, autologous nerve grafts, nerve allografts).

Cold sensitivity, and its related symptoms, often present in individuals with surgically treated neuroma as well as after a repaired or reconstructed nerve injury^54,55^, can be perceived as pain with an atypical painful response to cold^56–58^. Sensitivity to cold is also associated with worse sensory function^57^, and a higher disabling score (QuickDASH)^59^. Cold sensitivity correlated to total QuickDASH score and the other variables to an alterable extent, as well as it was associated with pain, which indicates the complexity that one symptom often come together with the other symptoms and disabilities^60,61^. In fact, neuropathic pain syndromes are heterogenous and multidimensional in their clinical entity, reflecting pathophysiologic mechanisms^46^. Concluded from the linear regressions, sleeping difficulties and ability to perform daily activities had an impact on pain both at rest and on load. Sleeping difficulties and ability to perform daily activity had also a high impact on total QuickDASH score, which indicate their importance for individuals’ quality of life. Interestingly, the postoperative follow up time was associated with a weak (negative) association with total QuickDASH score and pain on load, indicating that there is a minor improvement in symptoms and disability over time. In contrast, pain at rest and cold sensitivity did not improve over time, which is relevant information from the clinical perspective.

The study was based on two questionnaires, QuickDASH and HQ-8. QuickDASH consists of 11 questions on difficulties to perform regular activities, regardless of which limb that is used. HQ-8 on the other hand, consists of seven questions on symptoms in affected hand, and one on ability to perform daily activities^30^. The main difference between them is that injured side is taken in account in HQ-8^62^. However, these are non-disease specific and other potential diseases might interfere with the results. The use of other questionnaires for screening of neuropathic pain has also been stressed^63^. In the present study, a detailed analysis was not performed to evaluate any influence of co-morbidities. Neuroma treatment is an issue of “personalized medicine”, where all individual aspects should be considered in the treatment of the patient; aspects possibly also including analysis of potential biomarkers in plasma and tissue in the future^64–68^.

### Limitations and strengths

Despite an individual group with chronic pain that might be insufficiently treated, the response rate of 61% is consensual with other studies^69^. In accordance with previous studies, the non-responders were often men and younger, but, except for sex and age at surgery, there were no major differences between responders and non-responders in accordance with other follow up studies^62,70^. The questionnaires sent out were in Swedish, therefore non-Swedish speaking individuals were defined as non-responders. Furthermore, individuals with severe mental illnesses judged to be incapable of answering the questionnaires, and individuals with no contact information were not sent a questionnaire and therefore defined as non-responders. The number of included individuals can be perceived as low, but more than two thousand individual’s folders were read to identify the present individual group^37^. In accordance with other studies^71^, we did not anticipate that the incidence of nerve injuries changed over time in our regions. However, we cannot exclude that some individuals may be overseen by mistake. Identification of individuals, with such treated single neuroma, could not have been done in another way since there is no specific ICD-code for neuroma. In future studies, the use of ICD-codes for neuroma, for example in a national register, would facilitate the inclusion of individuals. The prevalence of neuroma remains unknown, but due to several used diagnose codes most surgically treated neuroma individuals at our hospitals should have been included. Individuals in the present study were surgically treated for some type of residual problems after their initial injury and any subsequent primary surgery, where the problems were interpreted and defined as a neuroma by the surgeon; thus, the indication for secondary surgery. A strength is the used questionnaires, QuickDASH and HQ-8, which are validated, and complement each other, where HQ-8 being more specific and QuickDASH contributing with a broader perspective of activity disabilities^30^.

## Conclusion

Individuals with a surgically treated neuroma may have extensive residual problems with pain and experience associated symptoms, like stiffness, weakness, numbness/tingling and cold sensitivity, which affect their daily activities, such as work, or sleep. Sleeping problems are associated with more limitations to work and to perform daily activities, as well as with extensive symptoms and disabilities. Surgery on a major nerve with a neuroma, compared to a digital nerve, yields more limitations to work or to perform other regular daily activities. Such limitations come along with a higher disability score, difficulties to sleep and more pain with associated symptoms. Despite surgery or method of surgery, individuals with a surgically treated neuroma experience persistent substantial symptoms, and disabilities, which affect their quality of life and with limited improvement over time. Therefore, it is crucial to prevent their appearance, but also it is important to do individual follow up so appropriate symptomatic treatment can be instituted.

## Data Availability

The complete and detailed individual data of all subjects cannot be publicly available for ethical and/or legal reasons due to compromising patient privacy based on the Swedish law. The National Ethical Committee (https://etikprovningsmyndigheten.se/en/) have imposed these restrictions. Data can be obtained after application and approval of the research project by the National Ethical Committee (https://etikprovningsmyndigheten.se/en/) and by the data safety committees of the regional health care systems in Region Skåne, Sweden (KVB-decision; https://vardgivare.skane.se/kompetens-utveckling/forskning-inom-region-skane/utlamnande-av-patientdata-samradkvb/) and from the appropriate unit in Region Östergötland, Linköping, Sweden.
